# A shared protocol for porcine surfactant use in pediatric acute respiratory distress syndrome: a feasibility study

**DOI:** 10.1186/s12887-019-1579-3

**Published:** 2019-06-18

**Authors:** Andrea Wolfler, Marco Piastra, Angela Amigoni, Pierantonio Santuz, Eloisa Gitto, Emanuele Rossetti, Carmine Tinelli, Cinzia Montani, Fabio Savron, Simone Pizzi, Luigia D’amato, Maria Cristina Mondardini, Giorgio Conti, Annalisa De Silvestri

**Affiliations:** 1 Division of Anesthesia and Intensive Care Unit, Department of Pediatrics, Children’s Hospital Vittore Buzzi, Via Castelvetro 32, 20152 Milan, Italy; 2 Pediatric ICU, Fondazione Policlinico Universitario A. Gemelli IRCCS, Università Cattolica del Sacro Cuore, Rome, Italy; 3 Pediatric ICU, Department of Woman’s and Child’s Health, University Hospital, Padova, Italy; 4 Department of Neonatal and Pediatric Intensive Care, Azienda Ospedaliera Universitaria Integrata, Verona, Italy; 5 Pediatric ICU, Pediatric Department, University Hospital G Martino, Messina, Italy; 6 Pediatric ICU, Department of Anesthesia and Intensive Care, Children’s Hospital Bambino Gesù, Rome, Italy; 7 Clinical Epidemiology and Biometric Unit – Foundation IRCCS San Matteo, Pavia, Italy; 8 Pediatric ICU, Department of Anesthesia and Intensive Care, Foundation IRCCS Ca Granda, Ospedale Maggiore Policlinico, Milan, Italy; 9 Pediatric ICU, Department of Anesthesia and Intensive Care, Institute for Maternal and Child health, IRCCS Burlo Garofolo, Trieste, Italy; 10 Pediatric ICU, Department of Anesthesia and Intensive Care, Children’s Hospital Salesi, Ancona, Italy; 11 Pediatric ICU, Department of Anesthesia and Intensive Care, Children’s Hospital Santobono-Pausillipon, Naples, Italy; 12 Pediatric ICU, Department of Pediatric Anesthesia and Intensive Care, University Hospital St. Orsola Malpighi Polyclinic, Bologna, Italy

**Keywords:** pARDS, Surfactant, Poractant, Infants, Pediatric intensive care unit

## Abstract

**Background:**

Pediatric ARDS still represents a difficult challenge in Pediatric Intensive Care Units (PICU). Among different treatments proposed, exogenous surfactant showed conflicting results. Aim of this multicenter retrospective observational study was to evaluate whether poractant alfa use in pediatric ARDS might improve gas exchange in children less than 2 years old, according to a shared protocol.

**Methods:**

The study was carried out in fourteen Italian PICUs after dissemination of a standardized protocol for surfactant administration within the Italian PICU network. The protocol provides the administration of surfactant (50 mg/kg) divided in two doses: the first dose is used as a bronchoalveolar lavage while the second as supplementation. Blood gas exchange variations before and after surfactant use were recorded.

**Results:**

Sixty-nine children, age 0–24 months, affected by Acute Respiratory Distress Syndrome treated with exogenous porcine surfactant were enrolled. Data collection consisted of patient demographics, respiratory variables and arterial blood gas analysis. The most frequent reasons for PICU admission were acute respiratory failure, mainly bronchiolitis and pneumonia, and septic shock. Fifty-four children (78.3%) had severe ARDS (define by oxygen arterial pressure and inspired oxygen fraction ratio (P/F) < 100**),** 15 (21.7%) had moderate ARDS (100 < P/F < 200). PO_2_, P/F, Oxygenation Index (OI) and pH showed a significant improvement after surfactant use with respect to baseline (*p* < 0.001 at each included time-point for each parameter). No significant difference in blood gas variations were observed among four different subgroups of diseases (bronchiolitis, pneumonia, septic shock and others). Overall, 11 children died (15.9%) and among these, 10 (90.9%) had complex chronic conditions. Two children (18.2%) died while being treated with Extracorporeal Membrane Oxygenation (ECMO). Mortality for severe pARDS was 20.4%.

**Conclusion:**

The use of porcine Surfactant improves oxygenation, P/F ratio, OI and pH in a population of children with moderate or severe pARDS caused by multiple diseases. A shared protocol seems to be a good option to obtain the same criteria of enrollment among different PICUs and define a unique way of use and administration of the drug for future studies.

## Background

Acute Respiratory Distress Syndrome (ARDS) represents a severe form of respiratory failure both for adults and children, with a lower prevalence (range 2.0–12.8%) and mortality (range 18–27%) in pediatric than in adult patients (range 17.9–81% and range 27–45% respectively) [1].

Many treatments have been used in pediatric ARDS (pARDS) with no clear preferred therapy [2]. This lack of convincing data has been stressed in a recent Consensus Conference conducted with the aim of identifying research priorities and develop recommendations regarding treatments of pARDS [3]. The authors stated that little is known about this condition, although areas of agreement were found among the experts. Surfactant is probably the therapy with the highest level of expectations, but still few convincing data support its use for pARDS. The rationale is that qualitative and quantitative deficiency of surfactant has a role in the development of acute respiratory failure [4, 5] and surfactant dysfunction is correlated with major clinical endpoints such as mortality and length of PICU stay [6]. On the other hand, the lack of large, randomized controlled trial (RCT) in pARDS and surfactant use limited the evidence supporting clear benefit. Most of the studies are on small series except three RCT on the effect of calfactant [7–9]. The populations enrolled are poorly homogenous for surfactant dosing, age or underlying diseases. The initial evidence suggested that surfactant could be more useful in primary ARDS such as respiratory infection, aspiration, trauma and near-drowning [10–12]. Indeed, in these conditions surfactant deficiency might be more relevant.

Studies on infants with ARDS related to respiratory syncytial virus (RSV) infection treated with surfactant showed an increase of ventilator-free days and a reduction of PICU length of stay (LOS) [13]. However, there is no clear preferred dose, route, timing and frequency of administration.

Finally, the type of surfactant used might be considered. Different exogenous surfactant are available for clinical use: porcine (poractant alfa), bovine (calfactant), synthetic (lucinactant). Not all showed the same results when used in vivo. Poractant alfa seems to be the one with the best efficacy in terms of oxygenation improvement while calfactant has shown conflicting results [7–9].

The aim of this retrospective multicentre observational study was to evaluate whether sharing a protocol on poractant alfa use in pARDS that specifies when to consider its use, the amount of drug, and the way of administration, might improve the benefits in terms of gas exchange in children less than 2 years old.

## Methods

A standardized protocol for surfactant administration developed by the Gemelli PICU was presented and shared within the Italian PICU network (TIPNet) in 2014. Two physicians from each center were responsible for data collection. During a specific course, the protocol was explained with frontal lessons and demonstrated through High-Fidelity (HI-FI) simulation. HI-FI simulation is an interactive training and learning methods using realistic clinical scenarios and interactive manikin.

Surfactant administration was suggested in children with acute respiratory failure (ARF), bilateral infiltrates, a P/F ratio less than 200 when mechanically ventilated with a plateau pressure <  30 cmH_2_O and a PEEP ≥5 cmH_2_O.

**Fig. 1:**
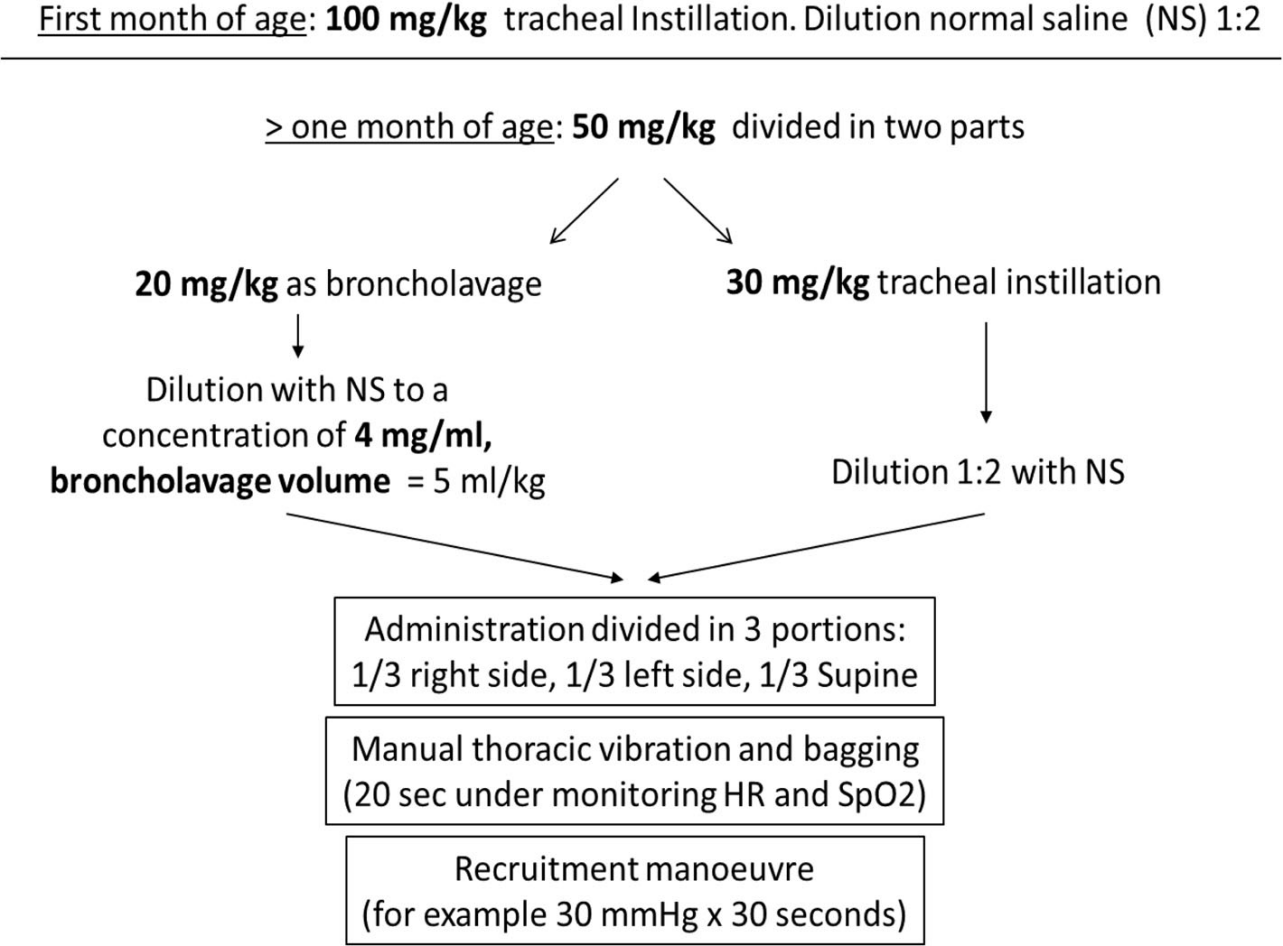
Administration protocol of exogenous surfactant

The protocol provided for the use of a surfactant dose of 100 mg/kg in infants less than 1 month of age administered as tracheal instillation. For older children, the dose was 50 mg/kg divided in two doses. The first dose, 20 mg/kg, was administered as lavage exploiting the detergent properties of surfactant. It was diluted with saline to obtain a concentration of 4 mg/ml of surfactant. Surfactant was administered in three aliquots, with the patient lying in three different positions: right side down, left side down and supine. After each aliquot, bagging is necessary to spread the drug as much as possible. Then, tracheal aspiration is mandatory to remove liquids and clear the airways. The second 30 mg/kg dose is administered as a supplementation within the first 2 minutes after recruitment by bagging and subsequent tracheal aspiration for lavage fluid recovery. It was diluted 1:2 with saline and administered as described above (Fig. 1). After both doses, lungs recruitment was carried out, e.g. 30 cmH_2_O for 30 s. During this second phase, tracheal aspiration was not performed for the first 2 h, to obtain the highest surfactant effects. Subsequent doses might be eventually administered following local clinical decision on each single patient.

All the Italian PICUs that shared the study protocol were invited to participate in the study. Each unit retrospectively collected data on children with age less than 2 years, affected by ARDS defined following the Berlin definition [14], mechanically ventilated, treated with surfactant between 1 September 2014 and 31 March 2017. Exclusion criteria were children with a limitation of intensive care treatment. The timing of drug use was based on the treating clinician’s choice.

Recruited patients were treated as per study protocol for the surfactant administration while all other treatments were as per standard practice at the study sites. All the centers had inhaled nitric oxide (iNO) and high frequency oscillatory ventilation (HFOV) available, while three centers had ECMO available in the same hospital.

Data collection consisted of patient demographics (age, gender, primary reason for PICU admission, comorbidity, PICU length of stay and PICU outcome), ventilator settings (ventilation mode, peak inspiratory pressure, PEEP, FiO_2_) and arterial blood gases (ABG) (pH, PaO_2_, PaCO_2_). The latter two were collected before and after surfactant administration and during the four subsequent days. The best ABG for each day was reported. When all required data were available, we calculated the oxygenation index (OI). OI is calculated as FiO_2_ x MAP/PaO_2_ where MAP is mean airway pressure.

The Local Ethical Committee of the Children’s Hospital Vittore Buzzi, reviewed and approved the study and waived informed consent due to the observational and retrospective nature of the study.

### Statistical method

*Power considerations:* looking at the data published in previous studies [15–17] we expected a clear effect of poractant alfa on blood gases. Using a repeated measures design, a group of 70 patients enrolled with at least 6 measures each could obtain a power higher than 95% in comparison of each time point versus baseline when the effect size is 0.79, corresponding to a standard deviation of the difference between time point means equal to 0.25.

*Statistical analysis*: categorical variables were described as count and percentage; quantitative ones.

as mean, standard deviation (SD) and Standard Error or median and Interquartile Range (IQR), as appropriate.

Primary end points were the changes in blood gases (PaCO_2_, PaO_2,_ PaO_2_/FiO_2_), OI and pH measured pre and post surfactant administration and in each of the four subsequent days; their changes before and after surfactant use were studied through analysis of variance for repeated measures. Multivariate models of analysis of variance for repeated measures were fitted to find associations between demographic or clinical factors and each blood gas or pH. Results are expressed as coefficient with their 95% confidence interval (CI) and presented with term specific *p*-values; the coefficient represents the mean variation of outcomes for unit change of quantitative predictors or between levels of categorical or ordinal predictors.

Secondary end-point was ARDS mortality during PICU stay. ARDS has been classified following Berlin definition in mild, moderate and severe. A univariate logistic regression has been tested between mortality and each variable considered in the study. Results are expressed as Odds Ratio (OR) and presented with 95% CI. The OR represents the odds that an outcome will occur given a particular exposure, compared to the odds of the outcome occurring in the absence of that exposure. For quantitative variables it represents the increase (or decrease) of risk for 1-unit change in independent variable. A multivariate analysis has not been made to evaluate if there were independent variables associated with survival due to the low number of deaths. *P* values < 0.05 were considered to be statistically significant. Data analysis was performed with STATA statistical package (release 15, 2017, Stata Corporation, College Station, Texas, USA).

## Results

Fourteen PICUs took part in the study which enrolled 71 children. Two patients were excluded as they died within 10 h after PICU admission, therefore 69 patients were analysed. Table 1 shows demographic characteristics. The number of patients enrolled from each unit ranged from one to nine with a mortality between zero and 50%.

**Table 1:** Description of the children enrolled in the study. Data are expressed as n (%) or otherwise indicated

Descriptive variables	Overall, *n* = 69	Death, *n* = 11 (15.9)	Survival, *n* = 58 (84.1)
Age, dd median (IQR)	115 (56.5–265)	158 (67.5–467.5)	109 (53.7–258.5)
Weight, kg median (IQR)	5 (4–7.2)	6 (4.6–7.45)	4.95 (4–7)
Gender (F/M)	1.5 (42/27)	1.2 (6/5)	1.6 (36/22)
Study year			
2014	18 (26.1)	3 (16.7)	15 (25.9)
2015	15 (21.7)	1 (6.7)	14 (24.1)
2016	25 (36.2)	5 (20)	20 (34.5)
2017	11 (15.9)	2 (18.2)	9 (15.5)
Comorbidity	27 (39.1)	10 (37.0)	17 (63.0)
Neurologic	6 (22.2)	1 (16.7)	5 (83.3)
Cardiac	11 (40.7)	5 (45.4)	6 (54.5)
Respiratory	6 (22.2)	3 (50.0)	3 (50.0)
Other	4 (14.8)	1 (25.0)	3 (75.0)
Preterm	17 (24.6)	2 (11.8)	15 (88.2)
Severe (< 30 w GA)	9 (52.9)	1 (11.1)	8 (88.9)
Moderate (30 < w GA < 35)	6 (35.3)	1 (16.7)	5 (83.3)
Late (35 < w GA < 38)	2 (11.8)	0	2
Origin			
Other hospital	32 (46.4)	7 (21.9)	25 (78.1)
Ward	15 (21.7)	2 (13.3)	13 (86.7)
ER	10 (14.5)	2 (20.0)	8 (80.0)
Other origin	12 (17.4)	0	12 (100)
Aetiology			
Medical	63 (91)	13 (20.6)	50 (79.4)
Surgical	4 (5.8)	0	4 (100)
Trauma	2 (2.9)	0	2 (100)
Underlying disease:			
Intrapulmonary	53 (76.8)	7 (13.2)	46 (66.7)
Extrapulmunary	16 (23.2)	4 (25.0)	12 (75.0)
LOS, dd median (IQR)	19 (14–26)	21 (15–31)	17.5 (14–25.75)
Mechanical ventilation			
Before PICU admission	25 (43.9)	5 (20.0)	20 (80.0)
On PICU admission	21 (36.8)	6 (28.6)	15 (71.4)
During PICU stay	11 (19.3)	0	11 (100)
Type of MV			
ETI	26 (40.6)	7 (26.9)	19 (73.1)
ETI + NIV	38 (59.4)	5 (13.2)	33 (86.8)

*dd* days, *IQR* interquartile range, *w GA* weeks of gestational age, *ER* emergency room, *LOS* length of stay, *ETI* endotracheal intubation, *NIV* non invasive ventilation

Pneumonia and bronchiolitis affected 22 (31.9%) children each, and 7 children had a sepsis-related diagnosis (10.1%). Four children developed pARDS during PICU stay after a surgical procedure for major congenital malformation while surfactant was used in two trauma patients. Among children with chronic complex conditions on admission, 11 infants (15.9%) had a congenital heart defect, 6 (8.7%) had a neurologic deficit, 6 (8.7%) had chronic respiratory disease, 4 (5.8%) were immunocompromised and 2 (2.9%) had congenital malformations. Mortality was higher in the respiratory group (3 deaths, 50%) and in the cardiological group (5 deaths, 45.4%). One patient died affected by a neurological syndrome. Seven infants had septic shock and 2 died (28.6%). Seventeen infants (24.6%) were born prematurely, of which 6 had chronic respiratory disease (bronchopulmonary dysplasia). The mean time between PICU admission and enrollment was 3.75 (± 4.1) days. Twenty-nine children (42.0%) received surfactant within 48 h of ARDS onset and two children (6.9%) died, while 32 (46.4%) were treated later, between 3 and 10 days from ARDS onset and 6 died (18.7%). In 8 patients (11.6%) (3 deaths, 37.5%) we could not establish the exact time between ARDS onset and surfactant use. The difference in terms of days between patients who died and those who survived was statistically significant (*p* < 0.05).

Fifty-four children (78.3%) had severe ARDS, 15 (21.7%) had moderate ARDS (100 < P/F < 200). Among the severe forms, 49 were classified with P/F and 5, due to the absence of arterial blood gas analysis, with SpO_2_/FiO_2_ less than 150. None of the enrolled children had a mild ARDS (200 < P/F < 300).

Nineteen children (27.5%) received iNO while 21 (30.4%) were ventilated with HFOV during the respiratory failure. Six children (8.7%) received ECMO. Almost all the patients were prono-supinated (*n* = 60, 86.9%) during PICU stay every 4 or 6 h, depending on local protocol.

**Fig. 2:**
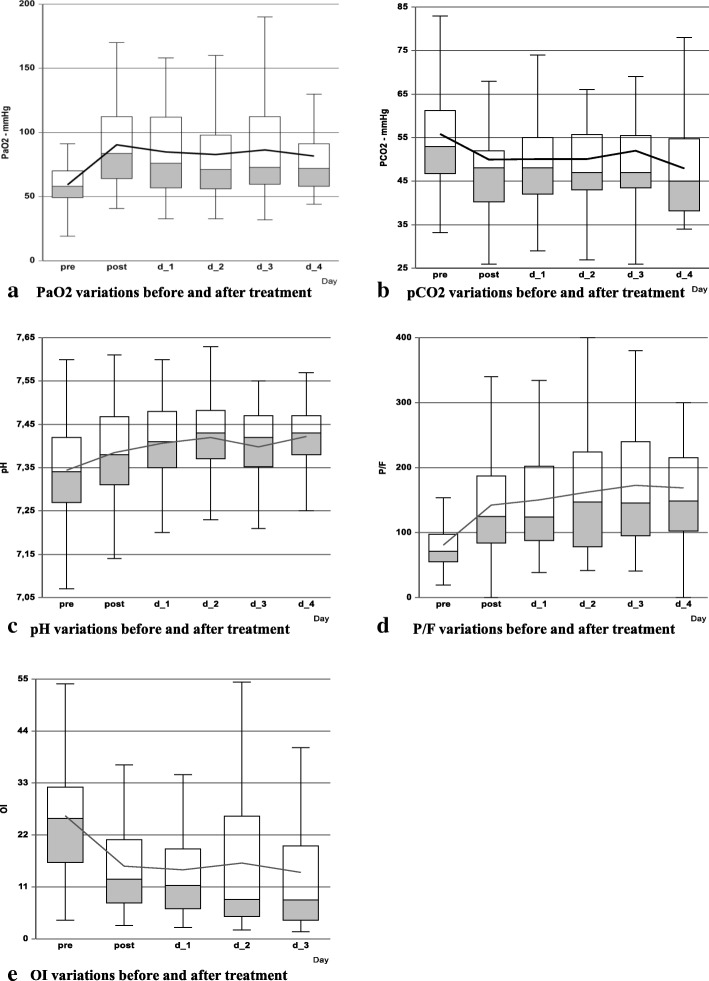
Blood gases variations immediately before and after surfactant administration. **a**: PaO2 variations before and after treatment. **b**: PaCO2 variations before and after treatment. **c**: pH variations before and after treatment. **d**: P/F variations before and after treatment. **e**: OI variations before and after treatment. Legend: Connecting line in each figure suggests the mean values. PaO_2_ = oxygen arterial pressure; PaCO_2_ = carbon oxide arterial pressure; P/F = oxygen partial pressure inspired oxygen fraction ratio; OI = Oxygenation Index; pre = before treatment; post = after treatment; d = day; OI data available only for 42 children

Blood gases are reported in Fig. 2 as box plot. For 42 children (60.9%) we might calculate OI. PaO_2_, P/F, pH and OI showed a significant improvement after surfactant use with respect to baseline (*p* < 0.001 at each included time-point for each parameter). Meanwhile, the manoeuvre did not increase PaCO_2_ which instead showed a significant reduction in all the observed period except on day 2.

**Fig. 3:**
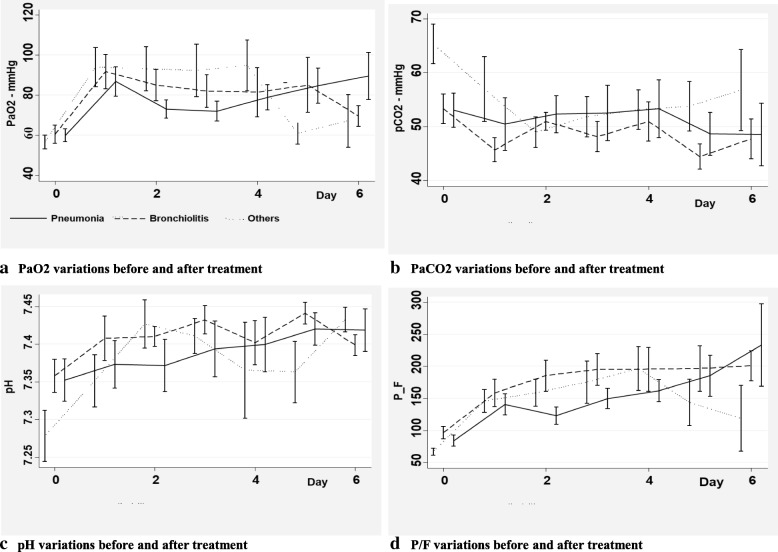
Blood gases variations in different subgroups of diagnosis immediately before and after surfactant administration. **a**: PaO_2_ variations before and after treatment. **b**: PaCO_2_ variations before and after treatment. **c**: pH variations before and after treatment. **d**: P/F variations before and after treatment. Legend: Data are expressed as means and standard errors. PaO_2_ = oxygen arterial pressure; PaCO_2_ = carbon oxide arterial pressure; P/F = oxygen arterial pressure inspired oxygen fraction ratio

We then analysed blood gases variations in three different subgroups of diseases (bronchiolitis, pneumonia, and others) to find out a possible response in specific subgroups. No significant differences were observed in the three subgroups of patients (Fig. 3).

As shown in Table 2, HFOV was associated with lower PaO_2_ and pH and higher PaCO_2_; prematurity and outcome were associated with PaO_2_ only.

**Table 2:** Factors associated with blood gases (PaO_2_, PaCO_2_) and pH variations pre and post surfactant administration and in each of the four subsequent days. Data are expressed as coefficients and 95% CI

Associated factors	PaO2	PaCO2	pH
Age	−0.06 (− 0.05–0.01)	0.00 (− 0.01–0.02)	0.00 (0.00–0.00)
CCC	10.05 (−3.70–23.80)	−1.97 (− 8.76–4.83)	−0.02 (− 0.06–0.02)
Prematurity	14.21 (2.42–26.02)*	0.72 (− 4.32–5.77)	−0.003 (− 0.05–0.04)
HFOV	− 16.48 (− 28.77 - -4.18)*	9.44 (2.29–16.59)*	−0.08 (− 0.13 - -0.02)*
iNO	−12.16 (− 24.60–0.27)	6.78 (− 0.18–13.75)	− 0.01 (− 0.07–0.05)
P-S	10.29 (−2.89–23.48)	−4.64 (− 12.87–3.59)	0.02 (− 0.06–0.10)
Time interval adm - surf	−0.02 (− 0.26–0.21)	0.19 (− 0.04–0.42)	−0.001 (− 0.00–0.00)
Outcome	−19.55 (− 34.28 - -4.84)*	5.60 (− 2.63–13.85)	−0.003 (− 0.09–0.09)

*CCC* Chronic Complex Condition, *HFOV* high frequency oscillatory ventilation, *iNO* inhaled nitric oxide, *P-S* prono supination, *adm* admission, *surf* surfactant dose; * = *p* < 0.05.

Multivariate models of analysis of variance for repeated measures were fitted to find associations between demographic or clinical factors and each blood gas or pH variations. Results are presented with term specific *p*-values; the coefficient represents the mean variation of outcomes for unit change of quantitative predictors or between levels of categorical or ordinal predictors.

Overall, 11 children died during PICU stay (15.9%) and among these, 10 (90.9%) had complex chronic conditions. All children who died had severe pARDS and mortality for this form was 20.4%. Two children (18.2%) died on ECMO. Six children died after resuscitation, two after withdrawal and one after withholding of therapy. The univariate logistic regression showed a strong association between mortality and chronic complex condition (OR 0.072; CI 0.014–0.383), HFOV (OR 5.5; CI 1.40–21.5) and time interval between the beginning of ARDS and the administration of surfactant (OR 1.11; CI 1.02–1.28); while the other variables were not significantly associated (Table 3).

**Table 3:** Analysis of variables associated with mortality (univariate logistic regression)

Associated variables	OR	CI	*p* value
Weight	1.072	0.84–1.37	0.583
Age	1.002	0.99–1.01	0.183
Gender, M	1.364	0.37–5.00	0.640
Immaturity	1.538	0.29–8.09	0.611
CCC	0.072	0.01–0.38	0.002
HFOV	5.5	1.40–21.52	0.015
ECMO	3	0.48–18.85	0.241
P-S	0.308	0.06–1.48	0.142
iNO	2.619	0.69–9.91	0.156
Time Interval adm – surf	1.142	1.02–1.28	0.025
PaCO_2_, pre administration	1.025	0.98–1.07	0.268
PaO_2_, pre administration	0.981	0.94–1.03	0.405
FiO_2_, pre administration	259.236	1.12–5989	0.045
P/F, pre administration	0.97	0.94–1.00	0.066
pH, pre administration	2.451	0.01–829.4	0.763
OI, pre administration	1.023	0.97–1.07	0.369

*OR* odds ratio, *CI* confidence interval, *M* male, *CCC* Chronic Complex Condition, *HFOV* high frequency oscillatory ventilation, *P-S* prono supination, *iNO* inhaled nitric oxide, *OI* oxygenation index, *d* day, *adm* admission, *surf* surfactant dose

## Discussion

Surfactant use has been studied for many years in its different forms. Although its long history, only few trials in pediatric patients were published and the results were not homogenous. This study reports the effects of porcine surfactant administered through a shared protocol among different PICUs on gas exchange in moderate and severe pARDS. It has some important differences from what has already been published.

The first difference is that we considered all the pARDS patients, regardless of the etiology. Porcine surfactant has been demonstrated to improve gas exchange and to be helpful in severe acute respiratory failure in infants affected by RSV bronchiolitis [15, 16]. Conversely, the study conducted by Tibby with bovine surfactant form [17] was conducted on RSV infections and did not demonstrate acute gas exchange improvements, despite an improvement in lung compliance. Others forms of surfactant failed to demonstrate positive effects in pARDS. In his trial Moller used bovine surfactant but no differences were shown either in terms of reduction of MV days or for PICU length of stay [18]. For the same endpoints, Thomas [19] using lucinactant did not report any difference between cases and controls. A separate mention deserves the two studies published by Willson on the use of calfactant. In his first trial published in 2005 [7], he showed a positive effect in terms of mortality and ventilator free days but not as PICU LOS. Unfortunately none of these data has been replicated in the second study in 2013 [8]. A more recent RCT published by Thomas on the use of calfactant in patients with leukemia/lymphoma or after hematopoietic stem cell transplantation and pARDS reported data that did not support the use of calfactant among this high mortality cohort to increase survival [9].

No prospective or retrospective studies on porcine surfactant has been published on pARDS originated by different etiologies. In our study RSV bronchiolitis represents one third of the cohort while the remaining children enrolled had pARDS due to pneumonia or different systemic diseases (sepsis, abdominal). The effect seems to be similar and to improve oxygenation in the whole cohort, suggesting that Poractant alfa might help to improve gas exchange in pediatric ARDS in less than 2 years old children, besides RSV infections.

The second difference is that the protocol we developed is mainly based on the exploitation of the two characteristic actions of surfactant. The first one is the lavage effect. It allows the removal of inflammatory mediators and cells debris, clearing alveoli and small bronchi [4]. It is preparatory for the second dose which is the drug dedicated to restore the inactivated or lacking endogenous surfactant. This is a new approach of surfactant use. All the other authors used a single or even more doses but all with the aim to replace rather than remove. This is true both in older and more recent studies [8, 15–17]. The third difference is the introduction of the recruitment manoeuvre after each administration. The importance of recruitment has been well demonstrated in adults [20, 21] while not yet proven in pediatrics [3]. Nevertheless in moderate and severe ARDS it might be helpful to open the lung and reduce the peak inspiratory pressure and the risk of barotrauma of the lung.

In our study, the dose of 50 mg/kg was selected based on the available experience on porcine surfactant use in severe bronchiolitis-induced ARDS, as reported in the Cochrane review published in 2015 by Jat and Chawla [13].

The administration of surfactant in different moments might increase the risk of severe desaturation, lung damage and endotracheal tube dislocation. No major side effect was observed in our cohort. Oxygen desaturation was a temporary effect and was always preventable trough bagging with 100% oxygen during the procedure.

Mechanical ventilation settings still remains a crucial point and HFOV use was associated with the lowest pO_2_ and pH and the highest pCO_2_ which means the most severe respiratory failure. Conventional and non-conventional techniques as well as iNO should be available to offer the best options to these seriously ill children.

Mortality was a secondary end point of the study. None of the patients with pARDS generated by bronchiolitis died while one needed ECMO, suggesting that RSV infection benefits most by surfactant use, as already showed in other studies. Although RSV infection might induce severe forms of respiratory failure, it is a diagnosis with low risk of mortality as considered by the Pediatric Index of Mortality (PIM) score. The overall mortality observed in this study was 15.9% and increased to 20.4% in severe forms. In the European study published on the Berlin definition of pARDS [14], mortality was 17.2% while in the severe form a 25% mortality was reported. In a very recent study on 708 children with pARDS, overall mortality was 18.3 and 33% died for severe forms [22].

In our cohort less than 10% of children received ECMO and within these, two died. The majority of children who died had a complex chronic condition, mainly respiratory and cardiological. The increase of comorbidity among PICUs admissions is a matter of fact [23] and should be considered as a variable when analyzing outcome data. Our data show a positive trend between the early use of surfactant and survival. Most of the children who died (7/11) were transferred from other hospitals, suggesting therefore that the use of surfactant might have been delayed because of the low experience of a general hospital. This result should make us consider surfactant not as a late rescue therapy but as an early pARDS treatment.

Both P/F and oxygenation index are good markers to classify and describe ARF. OI behind arterial oxygen partial pressure and inspired oxygen fraction has a third variable, the mean airway pressure. It might define how the patient is ventilated and the weight of mechanical ventilation measuring the oxygenation. In this study the choice of P/F rather than OI as a marker of respiratory failure and response to surfactant treatment was due to the non-standardization of ventilation parameters and the absence of inclusion criteria except the P/F value as defined by the Berlin definition on ARDS. However, data on ventilator settings allowed the calculation of OI in the majority of patients enrolled and showed significant improvements as the other blood gas values.

This is the first multicentre study that evaluates the effects of a shared protocol of surfactant administration and dosage. The need for a common behavior was raised by the Pediatric Acute Lung Injury Consensus Conference [3]. In this document, a panel of experts stressed the lack of evidence on how this drug should be administered.

This study has several limitations. The first is that it is not a randomized control trial. It is a retrospective study with only an interventional group. The difficulty to perform a RCT in pediatrics is high and the reasons are well known [24]. Moreover, most of the centers that enrolled and treated patients are confident with surfactant use and might not accept to waive its use in moderate or severe pARDS. However, on the basis of this preliminary study, we plan to submit to the collaborative PICU network the design of a prospective randomized controlled trial in order to reach a higher level of evidence on the efficacy of surfactant in pARDS. Beside surfactant use (timing, dose, administration mode), the protocol should strictly define and describe how to manage ventilation, nutrition, fluid and transfusion management in order to reduce possible confounding behavior. The second limitation is that we did not record consistently pulmonary mechanics variables such as compliance and resistance. These measures might better define changes in treated patients and help to identify responder and non-responders. Moreover, not all the children with ARDS during the study years were enrolled in the study. Through the Italian registry of PICU admission (TIPNet) we estimated that 10–30% of children who developed pARDS in any form of severity did not receive surfactant. Unfortunately, we cannot stratify for severity (mild, moderate, severe) as this information is not available in the registry for all the patients. However, we asked the centers to describe the local habit and in most of them surfactant is used only for severe pARDS forms. The protocol dissemination among PICU teams and the skills needed for surfactant use contributed to loss of some cases. A high variability in practices in different PICUs, has been recently published by Newth et al. [25]. This study showed how pediatric intensivists are inconsistent in their decisions about ventilatory support in children with pARDS and how ventilator management varies substantially in these children.

Finally we suggest to use porcine surfactant in infants and preschool children up to 2 years of age affected by moderate or severe pARDS whatever the aetiology, following the PARDIE definitions and mechanically ventilated with PEEP higher than 8 cmH_2_O and a plateau pressure less than 30 cmH_2_O. To define ARDS severity we suggest to use either OI or oxygenation saturation index (OSI) for those children without an arterial blood gas analysis. Recruitment manoeuvre should follow surfactant administration as well as pronation.

## Conclusions

In conclusion, our data showed that the use of Surfactant in its porcine form improves oxygenation, P/F ratio and pH without adverse events for the patient affected by moderate and severe pARDS caused by different etiologies. This study also supports the administration in two different doses, lavage and substitution, the use of recruitment manoeuvre after each one, as well as the early use once the ARF is requiring high pressure mechanical ventilation and elevated FiO2 or the use of nonconventional ventilation modes.

## Data Availability

The dataset used and/or analysed during the current study is available from the corresponding author on reasonable request.

## Abbreviations

ABG: Arterial blood gas
ARF: Acute respiratory failure
CCC: Chronic complex condition
CI: Confidence interval
ECMO: Extra corporeal membrane oxygenation
FiO_2_: Oxygen inspired fraction
HFOV: High frequency oscillatory ventilation
iNO: Inhaled nitric oxide
IQR: Interquartile range
LOS: Length of stay
MAP: Mean airway pressure
OI: Oxygenation index
P/F: Oxygen arterial pressure and inspired oxygen fraction ratio
pARDS: Pediatric acute respiratory distress syndrome
pCO_2_: Carbon oxide arterial pressure
PICU: Pediatric Intensive Care Unit
pO_2_: Oxygen arterial pressure
P-S: Prono supination
RCT: Randomized control trial
RSV: Respiratory syncytial virus
SaO_2_: Oxygen saturation
SD: Standard deviation
